# Prevention of Hypertensive Disorders of Pregnancy: a Novel Application of the Polypill Concept

**DOI:** 10.1007/s11886-016-0725-x

**Published:** 2016-05-21

**Authors:** J. L. Browne, K. Klipstein-Grobusch, A. Franx, D. E. Grobbee

**Affiliations:** Julius Global Health, Julius Center for Health Sciences and Primary Care, University Medical Center Utrecht, Heidelberglaan 100, 3584, CX Utrecht, The Netherlands; Division of Epidemiology and Biostatistics, School of Public Health, Faculty of Health Sciences, University of the Witwatersrand, Johannesburg, South Africa; Department of Obstetrics and Gynecology, University Medical Center Utrecht, Utrecht, The Netherlands

**Keywords:** Hypertensive disorders, Pregnancy, Polypill

## Abstract

Nearly all of the annual 287,000 global maternal deaths are preventable. Hypertensive disorders of pregnancy (HDP) are among the major causes. A novel fixed-dose combination pill or *polypill* to prevent cardiovascular disease is a promising strategy for prevention of HDP. The aim of this study was to identify eligible candidates for a polypill for the prevention of HDP. A comprehensive review of systematic reviews on drug and dietary interventions to prevent HDP was conducted. Interventions were evaluated based on efficacy, dose, route of administration, and side effects. Fourteen interventions were assessed. Low-dose aspirin and calcium were identified as candidates for a polypill, with risk reduction estimations for pregnancy-induced hypertension and preeclampsia ranging between 10 and 62 %, depending on patient population characteristics including a priori risk, and gestation age at start of intervention. Their effect may be augmented through the addition of vitamin D, vitamin B_12_, and folic acid. The effect and optimal composition needs to be evaluated in future trials. Given the persistent burden of maternal and perinatal mortality associated with HDP, prevention of these disorders is key—especially in low-resource settings. The polypill approach with a combination of aspirin, calcium, vitamin D, vitamin B_12_, and folic acid is a promising strategy to improve maternal and perinatal health outcomes.

**Electronic supplementary material:**

The online version of this article (doi:10.1007/s11886-016-0725-x) contains supplementary material, which is available to authorized users.

## Introduction

Nearly all of the annual 287,000 global maternal deaths are preventable [1]. Most of these occur in low- and middle-income countries (LMIC), and particularly in Sub-Saharan Africa and South Asia [1]. Although substantial progress has been made to reduce the maternal mortality ratio (MMR) with a 45 % decline since 1990, this still falls short of the Millennium Development Goal 5’s target of a reduction by 75 % [2]. Improving maternal health thus remains a global commitment through Sustainable Development Goal (SDG) 3.1 with the ambition to reduce the global MMR to less than 70 per 100,000 live births by 2030 [3].

One of the major causes of maternal mortality is hypertensive disorders of pregnancy (HDP) [1]. These include pregnancy-induced hypertension (PIH), preeclampsia, eclampsia, and HELLP syndrome, and are characterized by increasing morbidity and mortality [4]. Besides improving early diagnosis and initiation of appropriate treatment for PIH and preeclampsia [5, 6•], prevention of the disorders from occurring is an essential strategy to reduce morbidity and mortality—particularly in low-resource settings where availability of adequate care is limited. A number of interventions to prevent HDP have been previously described and include low-dose aspirin and calcium supplementation [7, 8••].

In the prevention of cardiovascular diseases, fixed-dose combination pills or *polypills* are currently explored as a novel strategy to simultaneously address various risk factors at once and facilitate optimal adherence [9–13]. Yet, the potential to combine the various strategies to prevent HDP in a single pill has not been explored. The same guiding principles as originally proposed for a polypill apply: a large preventative effect in all women at increased risk, several causal risk factors targeted at once, and reduction of these risk factors by as much as possible [11].

The objective of this review is to identify eligible candidates for a polypill for the prevention of hypertensive disorders of pregnancy through a comprehensive review of systematic reviews and meta-analyses.

## Methods

A search of systematic reviews and meta-analyses was conducted in Pubmed in October 2015 for interventions to prevent HDP. The search string for systematic reviews included a search of Cochrane Library, as recommended elsewhere [14, 15]. Supplement 1 includes the search strategy. The search and article selection were performed by a single reviewer (JB).

### Eligibility Criteria

All peer-reviewed systematic reviews and meta-analyses of randomized controlled trials (RCTs) of drug or dietary supplement interventions for the prevention of HDP were eligible for inclusion.

The following inclusion criteria were applied: the objective of the review should be primary prevention of HDP. Participants could either have the intention to conceive or be pregnant without any HDP at inclusion. Women with chronic hypertension were eligible for inclusion. The intervention was compared to a placebo, no treatment, or an alternative treatment. Systematic reviews and meta-analyses were excluded if they addressed behavioral change interventions, were not published in English, or assessed secondary prevention of a hypertensive disorder (e.g., magnesium sulfate for the prevention of eclampsia).

If several meta-analyses were available for the same intervention, the Cochrane meta-analysis was used, unless other articles addressed a specific population, included good quality trials not yet included in the latest Cochrane review that affected the estimated effect size, or were individual patient data meta-analyses. When articles by the same authors were published multiple times, the most comprehensive review was considered for inclusion. For updated versions of Cochrane reviews, only the most recent was considered.

### Outcome Measures

Our primary outcomes of interest were pregnancy-induced hypertension (PIH) or preeclampsia, as defined by the reviews’ authors. Definitions of PIH or preeclampsia usually were an elevated blood pressure of at least 140 mmHg systolic or 90 mmHg diastolic after 20 weeks of gestation, without or with significant proteinuria, respectively. Secondary outcomes were reported side effects.

### Assessment of Methodological Quality of Reviews

Articles were assessed if they adhered to the methodology published by the Cochrane Handbook of Systematic Reviews of Interventions [16]. For reviews that did not adhere to the Cochrane methodology, it was assessed whether a risk of bias evaluation was performed for primary articles.

### Data Extraction

Information from included articles was extracted using a standardized form on the following items: article, year of publication, number of RCTs included, publication years of included RCTs, number of participants, number of participants in trials with HDP outcomes, daily dose of intervention, start of intervention across trials, control group, estimated effect on PIH or preeclampsia in relative risk (RR) with 95 % confidence interval (95 % CI), whether HDP risk was a primary or secondary objective of the study, the quality of the evidence as reported by the authors of the study, and whether an assessment of the risk of bias was included in original articles.

### Data Synthesis

The results of the review were primarily descriptive to provide a comprehensive overview of drug or dietary supplement prevention strategies.

For each identified intervention, suitability for inclusion in a polypill was evaluated based on review reports on efficacy, dose, route of administration, side effects (acceptable/not acceptable/acceptable in high-risk only), and quality of evidence. Each category was color-labeled favorable, unfavorable or, ambiguous/intermediate for polypill inclusion.

## Results

**Fig. 1:**
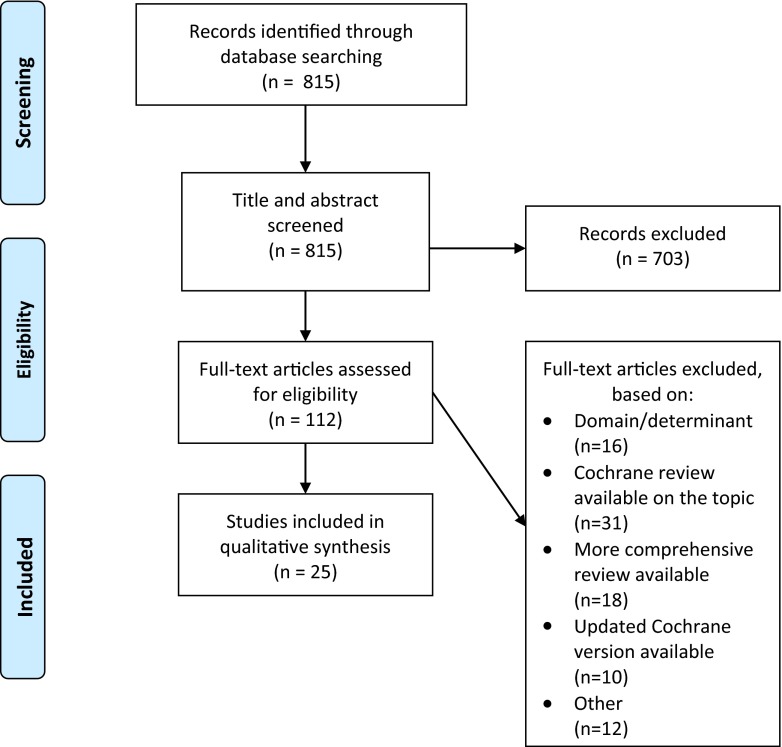
Flow chart of review process

After screening 815 articles identified in the search, 25 systematic reviews reporting on 14 drug and dietary interventions were included (Fig. 1). Table 1 includes an overview of the characteristics of included reviews.

**Table 1:**
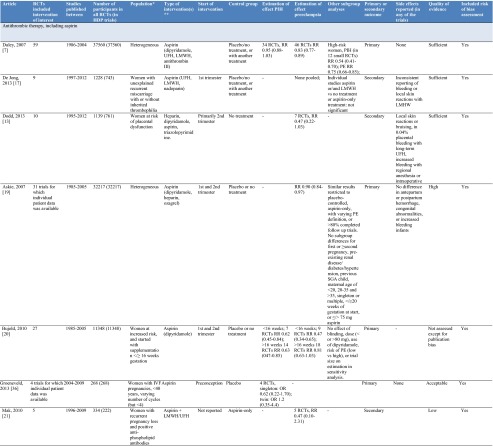

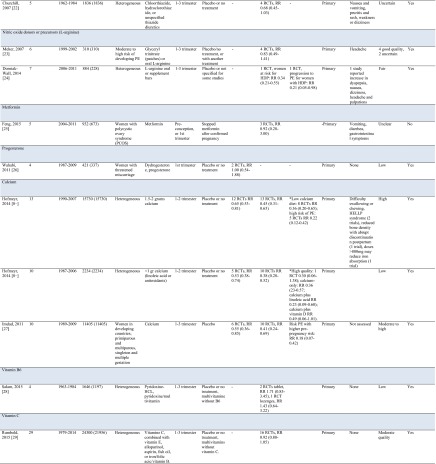

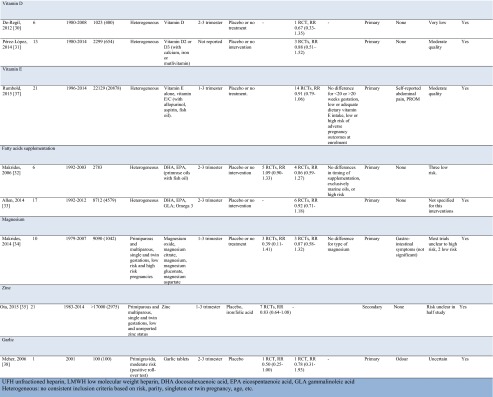
Overview of characteristics of included reviews

Medication and dietary interventions included antithrombotic therapy [7, 17–21, 36], nitric oxide (donors) [23, 24], diuretics [22], metformin [25], and progesterone [26].

Seven systematic reviews reported on antithrombotic therapy, primarily aspirin, for various populations; heterogeneous populations of pregnant women, women at risk of placental dysfunction and hypertensive disorders of pregnancy, women with unexplained recurrent miscarriages with or without inherited thrombophilia or positive anti-phospholipid antibodies, pregnant women after in vitro fertilization, and pregnant women who started with aspirin before 16 weeks of gestation [7, 17–21]. Based on an individual patient meta-analysis of more than 32,000 women, the estimated risk reduction for preeclampsia with aspirin supplementation is 10 % (RR 0.90; 95 % CI 0.84 to 0.97) [19]. The risk reduction is more than 50 % when aspirin is started before 16 weeks of gestation in women at risk of preeclampsia (9 RCTs, >11000 women, RR 0.47, 95 % CI 0.34 to 0.65) [20]. Doses of aspirin exceeding 75 mg/day may reduce the risk of preeclampsia more compared to lower doses [7], though no trial directly compared different doses. The combination of aspirin plus dipyridamole may further reduce the risk of preeclampsia (5 RCTs, 506 women, RR 0.30, 95 % CI 0.15 to 0.60) [7]. The estimated effects of aspirin or other antithrombotic interventions for specific populations of women at increased risk varied and were limited by small sample sizes.

Nitric oxide was evaluated in an unselected population of pregnant women and women with a moderate to high risk of developing preeclampsia [23, 24]. For high-risk women, a significantly reduced risk with the nitric oxide precursor l-arginine was observed (RR 0.34, 95 % CI 0.21 to 0.55). No risk reductions were observed with diuretics [22], metformin for women with polycystic ovary syndrome [25], and progesterone for women with threatened miscarriage [26].

Nutrient interventions were assessed in 11 systematic reviews and included calcium [8••, 27] vitamin B_6_ [28], vitamin C [29], vitamin D [30], vitamin E [37], fatty acids [32, 33], magnesium [34], zinc [35], and garlic [38],

Calcium supplementation with 1.5–2 g was assessed in a large meta-analysis (13 RCTs, >15000 women) and showed a reduction in the risk for gestational hypertension by 35 % (RR 0.65, 95 % CI 0.53 to 0.81) and preeclampsia by 55 % (RR 0.45, 95 % CI 0.31 to 0.65). The risk reduction for preeclampsia in women with a low calcium intake was 64 % (RR 0.36, 95 % CI 0.20 to 0.65) [8••]. In the same review, lower quality trials with a high risk of bias suggested that a daily supplement of less than 1 gram may reduce the risk of HDP (PIH 5 RCTs, RR 0.53, 95 % CI 0.38 to 0.74; preeclampsia 10 RCTs, RR 0.38, 95 % CI 0.28 to 0.58). Assessment of benefit of supplementation specifically in developing countries showed a 59 % reduction in preeclampsia (10 RCTs, RR 0.41, 95 % CI 0.24 to 0.69) among participating women [27]. The nitric oxide precursor l-arginine was observed in one RCT with 228 participants to reduce the risk of pre-eclampsia (RR 0.34, 95 % CI 0.21 to 0.55). None of the other nutrient supplements reduced the risk of HDP.

### Polypill Eligibility

In the assessment of interventions’ eligibility for inclusion in a polypill (Table 2), only aspirin and calcium remained after elimination of interventions for which there was no significant risk reduction for development of PIH or preeclampsia. An exception was l-arginine, which was excluded for the amount required (3–12 g), which exceeds tablets’ or capsules’ capacity.

**Table 2:**
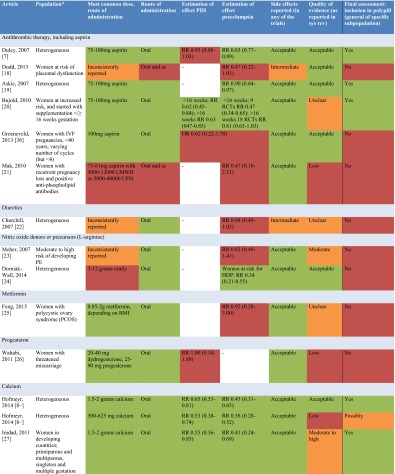

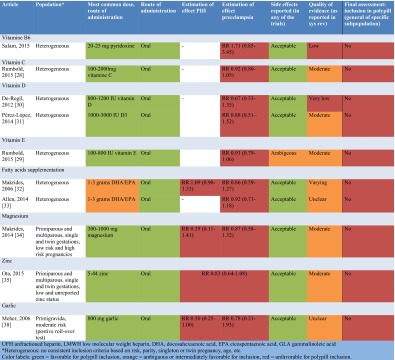
Assessment of interventions’ eligibility for inclusion in a polypill

Both aspirin and calcium were considered to have a favorable route of administration and required dose.

Side effects of both interventions appear acceptable given their beneficial effect. The concern that aspirin may increase antepartum hemorrhage, postpartum hemorrhage, or bleeding in children, was refuted by the individual patient analysis by Askie et al. based on 32,000 women that did not observe higher incidences [19]. For calcium, supplementation may result in mild gastrointestinal discomforts including difficulty swallowing and chewing. One significant concern with calcium supplementation was the observed increase in HELLP syndrome incidence (0.9/1000 vs 2.5/1000) in two trials. This was hypothesized to be the result of a masking effect with blood pressure lowering without addressing the underlying pathophysiologic process of preeclampsia when supplementation started in the second trimester, and caused delay in recognition, diagnosis, and treatment [8••]. Another concern with higher calcium supplementation (>800 mg) is a possibly reduced capacity for iron absorption [8••], particularly relevant given the persistent high global prevalence of (iron-dependent) anemia in pregnant women [39].

## Discussion and Conclusions

Based on a large body of evidence from randomized controlled trials, eligible candidates for a polypill to prevent hypertensive disorders of pregnancy are calcium and aspirin.

As this review only assessed potential preventative interventions evaluated in RCTs, a number of interventions for which no or too few high-quality trials exist were not included. For example, folic acid and vitamin B_12_ have been associated with a lower risk of preeclampsia in observational studies and are currently explored in clinical trials [40, 41]. Likewise, we did not include behavioral interventions in this review although effective interventions have been described and include diet and lifestyle-based metabolic risk modification [33].

As of yet, only one small trial (*n* = 49) has been conducted to evaluate the effect of aspirin and calcium simultaneously for the prevention of superimposed preeclampsia in women with chronic hypertension between 20 and 27 weeks of gestation [42]. This trial did not take a polypill approach, but instead provided aspirin pills and calcium carbonate powder to be dissolved in water, which may explain low adherence in the intervention arm (<50 % with optimal adherence). There was a non-significant lower rate of superimposed preeclampsia (52.2 vs 73.1 %, *p* = 0.11) in the intervention arm. One of the key advantages of the polypill approach is the improvement of adherence, especially when otherwise the pill burden would be high [11, 12].

The polypill approach limits the number of pills patients need to take to have an effective risk reduction. As the current WHO recommended dose of 1.5 to 2 g of calcium [43] requires multiple capsules or tablets, the growing evidence that risk reduction may also occur with lower amounts of calcium (as low as 500 to 600 mg) is encouraging and will enhance the feasibility of a polypill intervention [8••]. A further advantage of the polypill approach is that without an increase in the pill burden, and therefore with maintained or improved adherence, other components to support the effect of the primary intervention can be easily added.

We propose a polypill for the prevention of HDP consisting of calcium and aspirin. These combined interventions target different pathophysiologic pathways of HDP at once. Aspirin inhibits the results of preeclampsia-associated placental damage, with platelet and clotting system activation, and restores the imbalance between the vasoconstrictor thromboxane A2 and vasodilator prostacyclin [4, 7]. Calcium attenuates the effects of relatively low serum calcium levels on blood pressure: parathyroid hormone (PTH) and renin release with the consequences of increased levels of intracellular calcium, vasoconstriction of vascular smooth muscle, and increased peripheral resistance [8••].

The addition of three other components may enhance the polypill’s effect. Calcium resorption in the gut is enhanced through the addition of vitamin D. Folic acid and vitamin B_12_, based on the circumstantial evidence of observational data and previously hypothesized mode of action, reduce the risk of HDP further through improved placental and endothelial function by lowering plasma homocysteine levels [40, 41],

### Who Should Take the Polypill to Reduce Hypertensive Disorders of Pregnancy?

The polypill will be most effective for those at increased risk of developing HDP. However, the ability to accurately predict which women are at highest risk is hampered by the lack of robust prognostic models [4, 44, 45]. Still, risk factors are well described and increased risk for the development of preeclampsia established for women who are older, nulliparous, have antiphospolipid antibodies, preexisting diabetes mellitus, preexisting hypertension, multiple pregnancy, a higher BMI, and a pregnancy (family) history of hypertensive disorders [44, 46]. Given the likely very low rate of adverse events associated with the polypill components and the advantages of preventing maternal and perinatal morbidity and mortality associated with HDP, the prescription of the polypill to women from moderate risk upwards, i.e., those with the aforementioned risk factors, can be justified.

The polypill approach allows the development of alternative fixed-dose compositions designed for specific subgroups. For example, for women with preexisting hypertension or pregnancy-induced hypertension who require oral antihypertensive treatment with the beta-blocker labetalol, alpha-agonist methyldopa, or calcium channel blocker nifedipine [47–49], these drugs could be combined with aspirin or calcium in a polypill to reduce the risk of progression into severe HDP. Similarly, women with gestational diabetes without access to or contraindication for insulin may benefit from the addition of metformin [50, 51]. However, although metformin is a promising alternative to insulin, more randomized evidence about its effectiveness is required.

Importantly, the exact health impact and optimal composition of a polypill in pregnancy to prevent HDP for women at increased risk or specific subpopulations needs to be established in properly conducted randomized controlled trials. Ideally with multiple arms to explore various composition modalities, at various gestational ages, and in different populations including countries with limited resources. Subsequently, implementation studies will need to assess the cost-effectiveness and optimal integration in existing health systems.

Given the persistent burden of maternal and perinatal mortality globally associated with hypertensive disorders of pregnancy, prevention of these disorders from occurring is key—especially in low-resource settings. In addition, collateral wins are to be expected as calcium supplementation and low-dose aspirin have been associated with reductions in prematurity, intrauterine growth retardation, small for gestational age babies, stillbirth, and neonatal mortality [7, 8••, 19].

A polypill approach with a combination of aspirin, calcium, vitamin D, vitamin B_12_, and folic acid is a promising, safe, and effective strategy to promote improved maternal and perinatal health outcomes.

## Electronic Supplementary Material

Below is the link to the electronic supplementary material.

ESM 1 (DOCX 47 kb)
