# Supplementary material for: Prevention of Hypertensive Disorders of Pregnancy: a Novel Application of the Polypill Concept
Source: Curr Cardiol Rep. 2016 May 21;18:59. doi: 10.1007/s11886-016-0725-x (PMC4875943; doi:10.1007/s11886-016-0725-x)
Supplement: Supplementary file 1 — (DOCX 47 kb) [file 11886_2016_725_MOESM1_ESM.docx]

Supplementary file 1: Search strategy for interventions to prevent hypertensive disorders of pregnancy

Search ((((Cochrane Database Syst Rev [TA] OR search[Title/Abstract] OR meta-analysis[Publication Type] OR MEDLINE[Title/Abstract] OR (systematic[Title/Abstract] AND review[Title/Abstract]))))) AND (((("hypertensive disorders"[Title/Abstract]) AND pregnancy[Title/Abstract])) OR (((((preeclampsia[Title/Abstract]) OR eclampsia[Title/Abstract]) OR "gestational hypertension"[Title/Abstract]) OR "pregnancy induced hypertension"[Title/Abstract]) OR "hellp syndrome"[Title/Abstract]))
